# Twenty year experience of the oral rabies vaccine SAG2 in wildlife: a global review

**DOI:** 10.1186/s13567-014-0077-8

**Published:** 2014-08-10

**Authors:** Philippe Mähl, Florence Cliquet, Anne-Laure Guiot, Enel Niin, Emma Fournials, Nathalie Saint-Jean, Michel Aubert, Charles E Rupprecht, Sylvie Gueguen

**Affiliations:** Virbac, 13ème Rue LID, BP 27, 06511 Carros Cedex, France; Anses, Laboratory for Rabies and Wildlife, European Union Reference Laboratory for rabies, European Union Reference Laboratory for rabies serology, OIE Reference Laboratory for rabies, WHO Collaborating Centre on Research and Management on Zoonoses Control, Technopôle agricole et veterinaire, CS 40009, 54220 Malzeville, Cedex, France; Conseils en Pharmacie et Biologie, Sainte Foy les, Lyon, France; Veterinary and Food Board of Estonia, Väike Paala str. 3, 11415 Tallinn, Estonia; Chemin des Maures, 83440 Callian, France; Ross University School of Veterinary Medicine, Basseterre, St. Kitts West Indies

## Abstract

The SAG2 vaccine (RABIGEN® SAG2) is a modified live attenuated rabies virus vaccine, selected from the SAD Bern strain in a two-step process of amino acid mutation using neutralizing monoclonal antibodies. The strain is genetically stable and does not spread in vivo or induce a persistent infection. Its absence of residual pathogenicity was extensively demonstrated in multiple target and non target species (such as wild carnivores and rodent species), including non-human primates. The efficacy of SAG2 baits was demonstrated according to the EU requirements for the red fox and raccoon dog. The use of safe and potent rabies vaccines such as SAG2 largely contributed to the elimination of rabies in Estonia, France, Italy and Switzerland. Importantly, these countries were declared free of rabies after few years of oral vaccination campaigns with SAG2 baits distributed with an appropriate strategy. The excellent tolerance of the SAG2 vaccine has been confirmed in the field since its first use in 1993. No safety issues have been reported, and in particular no vaccine-induced rabies cases were diagnosed, after the distribution of more than 20 million SAG2 baits in Europe.

## Table of contents

1. Introduction
2. Main characteristics of the SAG2 vaccine
  2.1 Development of the SAG2 strain
  2.2 Genetic stability
  2.3 SAG2 bait
  2.4 Resistance of SAG2 bait and thermostability

3. Pathogenicity of the SAG2 strain in wildlife
  3.1 Target species
  3.2 Non target species

4. Immunogenicity and efficacy in wildlife animals in controlled laboratory trials
  4.1 Red Fox
  4.2 Raccoon dog
  4.3 Jackal
  4.4 Arctic fox
  4.5 Raccoon and skunk
  4.6 Mongoose
  4.7 Coyotes
  4.8 Ethiopian wolf

5. Efficacy of SAG2 in the field
  5.1 Switzerland
  5.2 France
  5.3 Estonia
  5.4 Italy
  5.5 Finland

6. Conclusions
7. Competing interests
8. Authors’ contributions
9. Acknowledgements
10. References

## 1. Introduction

Rabies is an acute progressive fatal viral encephalitis, caused by negative stranded RNA viruses in the Genus *Lyssavirus* (Family *Rhabdoviridae*, Order *Mononegavirales*). The disease is widely distributed on all continents except Antarctica [1].

In the early 1960s, wildlife emerged as the main reservoir for rabies virus in many developed countries. During the last decade, more than 90% of rabies cases occurring in Europe were reported in wildlife [2]. Until the 1970s, the control of wildlife rabies relied upon population reduction of rabies reservoir species by trapping and hunting foxes or other carnivores, gassing fox dens or distributing poisoned baits [3–6]. These measures proved ineffective in reducing rabies prevalence. An alternative strategy was considered, consisting of wildlife immunization using oral vaccine baits [7]. For the first time, the feasibility of vaccinating wildlife was demonstrated in Switzerland during 1978 using modified live rabies virus vaccines [8].

Most modified-live rabies virus vaccines used for oral vaccination (OV) originated from the attenuated Evelyn Rokitnicki Abelseth (ERA) virus strain, which was derived from the original Street-Alabama-Dufferin (SAD) rabies virus strain. The parental SAD strain was isolated from the salivary glands of a rabid dog in the USA during 1935, which was passaged in mice, chick embryos, and various cell lines and was re-named ERA [9]. The SAD Bern strain is a cell-adapted derivative of the ERA strain, and was used for the first trials of OV in Switzerland [10].

The SAD B19 and SAD 5/88 strains were derived from the SAD Bern by selection on cloned BHK21 cells [11]. However, SAD Bern, SAD B19 and SAD P5/88 retained a non negligible residual pathogenicity in rodents and in wild and domestic carnivores, even by the oral route [12–15]. Thus, using those strains in the field, there is a potential risk for contamination of target and non-target animal species during OV campaigns (for review, see the recent paper of Hostnik et al. [16]). The SAG2 strain (SAD Avirulent Gif), is a modified live virus strain selected from SAD Bern during 1990 by two successive mutations [17]. All the three strains (SAD B19, SAD Bern and SAG2) are currently used for OV of wildlife in Europe [18]. A recombinant vaccinia virus and a recombinant human adenovirus vector, both expressing the rabies glycoprotein of the ERA strain (V-RG and ONRAB, respectively) are also available. Those vaccines are mainly in use in the USA [19] and Canada [20].

Given its long history of safe and effective use in the field, this paper reviews the process development and main biological properties of the SAG2 strain, together with the experience of rabies control programmes undertaken with this vaccine in different European countries.

## 2. Main characteristics of the SAG2 vaccine

### 2.1 Development of the SAG2 strain Figure 1

**Figure 1:**
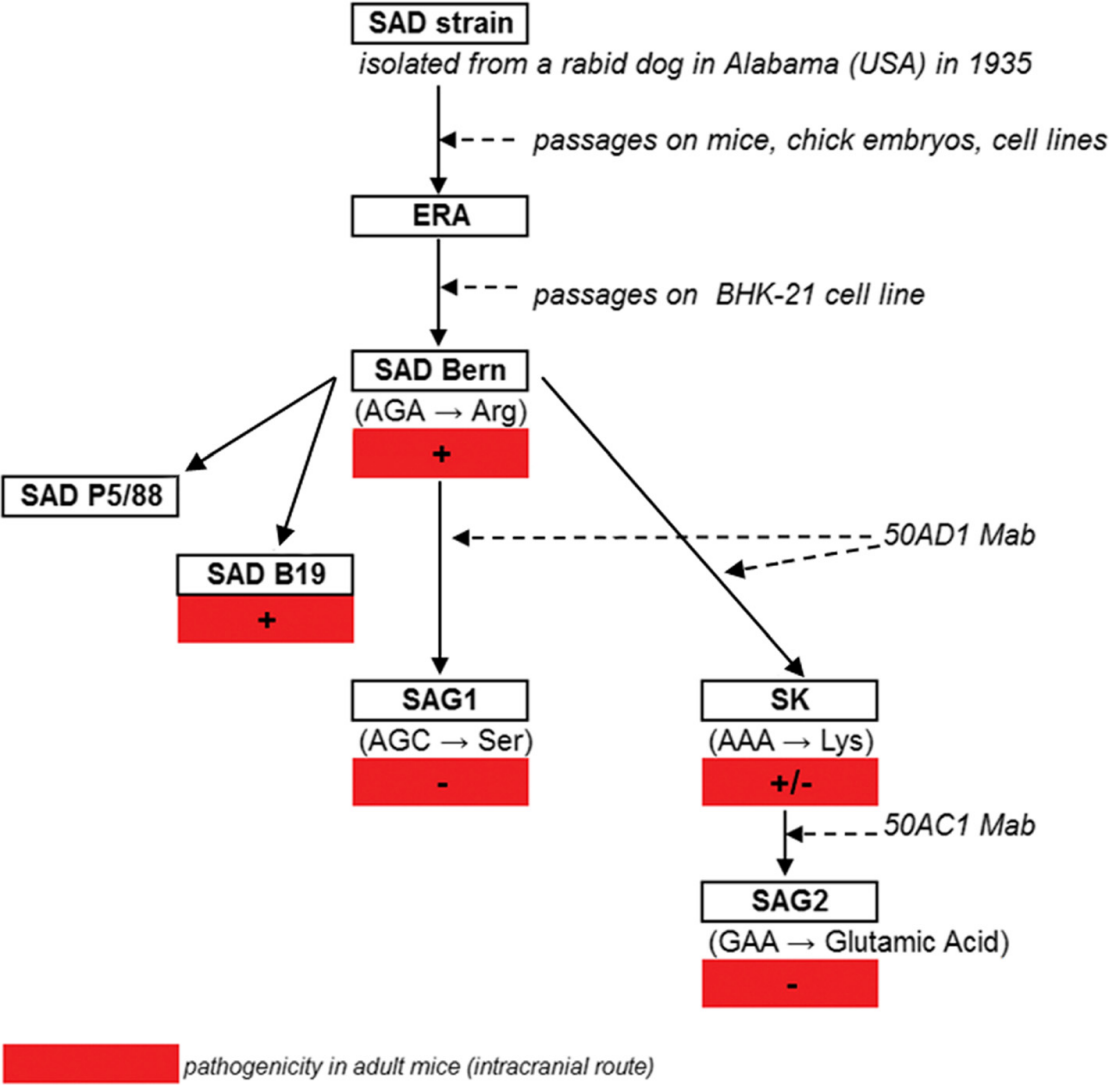
**Construction of the SAG2 strain.** ERA = Evelyn Rokitnicki Abelseth, Mab = monoclonal antibody. The parental SAD strain was isolated from the salivary glands of a rabid dog in the USA during 1935, which was passaged in mice, chick embryos, and various cell lines and was re-named ERA (Evelyn Rokitnicki Abelseth). The SAD Bern strain is a cell-adapted derivative of the ERA strain. The SAD Bern strain was cultivated in the presence of monoclonal antibodies binding specifically to one of the two major antigenic sites (antigenic site III) of the rabies virus glycoprotein, involved in virus pathogenicity. Under the selective pressure of these monoclonal antibodies, only variants of SAD Bern bearing an amino-acid substitution at the critical position 333 of the rabies virus glycoprotein escaped neutralisation in culture. An avirulent mutant, SAG1 (for SAD Avirulent Gif), in which arginine at position 333 was substituted by serine, was isolated from SAD Bern with monoclonal antibody (Mab) 50 AD1. The SAG2 strain was constructed from SAD Bern in a two-step selection procedure using neutralizing monoclonal antibodies. First, a mutant strain (SK) was selected from SAD Bern, where the arginine at position 333 was replaced by lysine. SAG2, a non pathogenic mutant resistant to neutralisation by monoclonal antibody 50 AC1 was selected from SK, where the lysine at position 333 was replaced by a glutamic acid. Thus, SAG2 can be considered as a double avirulent mutant, since the codon GAA, which codes for glutamic acid, differs from the codon AGA from SAD Bern (coding for arginine) by two nucleotides.

The World Health Organization (WHO) organized several meetings with rabies experts to define safety and efficacy requirements for OV use in wildlife and in dogs [3–6]. One approach, using hybridoma technology, was used to ensure the safety of the rabies virus vaccine strain, based upon the concept of selective antigenic variants [21]. The SAD Bern strain was cultivated in the presence of monoclonal antibodies binding specifically to one of the two major antigenic sites (antigenic site III) of the rabies virus glycoprotein, which is involved in virus pathogenicity. Under the selective pressure of these monoclonal antibodies, only variants of SAD Bern bearing an amino-acid substitution at the critical position 333 of the rabies virus glycoprotein escaped neutralisation in culture [22].

An avirulent mutant, SAG1 (for SAD Avirulent Gif), in which arginine at position 333 was substituted by serine, was isolated from SAD Bern with monoclonal antibody 50 AD1 [14,23,24]. This mutant was found to be avirulent in all animal species tested and in adult mice, regardless of the dose and route of inoculation [12,14]. The SAG1 virus was shown to be as immunogenic as the parental strain of SAD Bern. The substitution of arginine at position 333 improved considerably the safety of the vaccine strain without impairing its efficacy [23,24]. Since the frequency of spontaneous mutations in RNA viruses has been estimated to be about 10^-4^ [25], there was a theoretical, albeit limited, risk of reversion to a different phenotype which might possibly affect pathogenicity (equivalent to SAD Bern). To improve the genetic stability of the vaccine and upon request of the regulatory authorities, a double avirulent vaccine was developed with the Centre National de la Recherche Scientifique (CNRS, France). The SAG2 strain was constructed from SAD Bern in a two-step selection procedure using neutralizing monoclonal antibodies [17]. First, a mutant strain (SK) was selected from SAD Bern, where the arginine at position 333 was replaced by lysine. The SK strain was resistant to monoclonal antibody 50 AD1 and partially resistant to monoclonal antibody 50 AC1. The SK strain was avirulent when injected intramuscularly in adult mice and had an extremely low pathogenicity by the intracerebral route [17]. A mutant resistant to neutralisation by monoclonal antibody 50 AC1 was selected from SK, where the lysine at position 333 was replaced by a glutamic acid. This mutant, called SAG2, was non-pathogenic in adult mice, even by the intracerebral route [17]. Thus, SAG2 can be considered as a double avirulent mutant, since the codon GAA, which codes for glutamic acid, differs from the codon AGA from SAD Bern (coding for arginine) by two nucleotides [17,22–24].

### 2.2 Genetic stability

The laboratory mouse is one of most susceptible species to rabies virus, and the parental strain SAD Bern is highly pathogenic for adult mice by the intracerebral route [14]. Therefore, studies on reversion to virulence were conducted in mice. The model selected for serial passages was the suckling mouse. Suckling mice do not possess a sufficiently mature immune system to control rabies virus infection in the central nervous system and they are, for other reasons, particularly receptive to rabies virus multiplication. Five series of five consecutive intracerebral passages were conducted in suckling mice. At each passage, brain homogenates of the suckling mice were inoculated intracerebrally to adult mice. None of the adult mice developed rabies and no rabies virus antigens were detected in their brains. No pathogenic revertants were detected from these passages [26].

After 10 consecutive passages in BSR cells (a clone of BHK-21 cells), the SAG2 virus was injected intracerebrally to five adult mice. No deaths were recorded. The nucleotide sequence of the site III region of the virus stock was checked. Only the glutamic acid in position 333 was detected [17].

The genetic stability of the SAG2 strain was thus demonstrated in vivo (mice) and in vitro. Significantly, the SAG1 strain was already very stable, since only one of 18 adult mice died after intracerebral inoculation, following the first passage in suckling mouse brain, and none died after the second and third passages.

### 2.3 SAG2 bait

**Figure 2:**
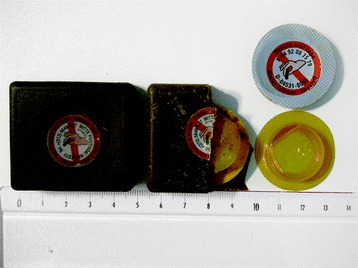
**SAG2 Vaccine bait.** This figure shows the bait, the PVC/aluminium blister containing the liquid suspension of the SAG2 strain. SAG2 bait and the blister are both labelled with “Rabies vaccine, do not touch”.

A liquid suspension of the SAG2 strain, with a titer ≥ 10^8^ CCID_50_/dose (CCID_50_ = Cell Culture Infective Dose 50%) is contained in a PVC/aluminium blister which is embedded in a bait. The bait is made from a mixture of fat as the vehicle, fish meal and natural fish aroma for palatability, paraffin for shape and protection against moisture, a polymer to provide mechanical resistance needed for dropping from the aircraft or helicopter, and tetracycline (150 mg/bait) as a biological marker for uptake. The size of the SAG2 bait is 4.9 × 4.4 × 1.5 cm and its maximum weight is 28.1 g (Figure 2). Vaccine baits are stored in a freezer between −40 °C to −20 °C and protected from light.

Since baits are released in the environment and may be touched by humans, SAG2 bait and blister are labelled with “Rabies vaccine, do not touch” (Figure 2). In addition, a phone number is provided on the blister and the bait, to provide information to persons who may find a bait by accident.

Since the risk of rabies cannot be eliminated completely, post-exposure prophylaxis (PEP) is necessary in case of contact with the SAG2 vaccine (WHO category II of human contact: nibbling of uncovered skin, minor scratches or abrasions without bleeding [27]). The impact of OV campaigns on the uptake of vaccine baits and contamination of people was assessed in France during 1992-1993. During this period, 4.4 million vaccine baits were distributed. A total of 71 people found and handled a bait (48 had a contact with a SAG1 bait and 23 with a V-RG bait) and 38 received PEP (31 for SAG1 and 7 for V-RG). No clinical consequences or serious incidents were recorded [28].

### 2.4 Resistance of SAG2 bait and thermostability

Since most baits are dropped from aircraft, it was important to demonstrate the mechanical resistance of the SAG2 bait. No damage was observed in SAG2 baits distributed by helicopter onto solid ground either at a speed of 180 km/h and an altitude of 60 to 100 m, or at a speed of 160 km/h and an altitude of 130 m [26].

Baits are resistant in water and not degraded by rainfall. By plunging baits into water for 4 days at 25 °C, a negligible weight gain of 0.2% and partial bleaching were observed [26]. The melting point of the bait is 56 °C [26]. Baits can be stored without any significant change in volume, pH, sterility and virus titer at −20 °C and −40 °C for 2 years, and at 4 °C or 25 °C for up to 7 days [26], following the recommendations of the EDQM (Pharmacopoeia) and EC [29].

The thermostability of the SAG2 vaccine has been confirmed in the field. The stability of the liquid form of the SAG2 strain placed in chicken head baits (such baiting highly appetent for fox is no more used in Europe) was tested in Zimbabwe: the titre decreased by l log_10_ CCID_50_ within 3 days (at sunshine temperatures in July and October ranging from 25 °C to 30 °C) when the chicken head baits were placed under grass or soil, but underwent marked titre loss when baits were placed in the sun or shade without covering vegetation [30]. Through a FAIR project (CT 97-3515 “Wildlife vaccination against rabies in difficult and emergency situations and its potential impact on the environment”), different trials were conducted during 1999 and 2000 in Belgium, France, Germany and Italy to assess the stability of all vaccine baits (V-RG, SAG2, SAD B19, SAD P5/P88) available in the EU [29]. At temperatures < 30 °C, which are usually observed when baits are distributed during spring and autumn, vaccine titres remained stable during a 3-week observation period for all baits. In contrast, titre losses were significant for all attenuated vaccines when temperatures exceeded 30 °C. In addition, the bait casing of SAD B19 and SAD P5/88 disintegrated more or less completely when exposed to high temperatures (30-35 °C) and rain. The bait casing of the V-RG was more stable, and the SAG2 casing showed an intermediate resistance [29].

## 3. Pathogenicity of the SAG2 strain in wildlife

A complete absence of pathogenicity of the SAG2 strain was demonstrated in adult mice when administered by various routes and doses [17]. The safety of the SAG2 strain by the oral route was confirmed in the dog and the cat when administered at a high (10^9^ PFU) dose [31].

### 3.1 Target species

The safety of the SAG2 strain in the fox and raccoon dog (Table 1) was assessed under controlled laboratory conditions before release in the field according to the requirements of the EDQM [32] and recommendations of WHO [27]. In most studies, the SAG2 strain was administered in a liquid form by direct instillation into the mouth to ensure that the entire dose was administered.

**Table 1:** **Summary of the safety data after oral administration of high doses of SAG2 vaccine to foxes and raccoon dogs**

**Species**	**No of animals, route (mean age)**	**SAG dose per animal**	**Time of observation**	**Specific morbidity/mortality**	**Virus isolation from saliva swabs**	**Rabies virus positive brains**	**Rabies virus positive salivary glands**	**Seroconversion**	**Source**
Fox	10 p.o. (6 months to 2.5 years)	10^9.8^ CCID50	183 days	0/10	0/10 (D0, D1, D3, D7)	0/10	0/10	10/10	[26]
	10 p.o. (9 months)	10^9^ p.f.u.	175 days	0/10	0/10 (D1, D3, D6)	0/10	0/10	10/10	
Raccoon dog	5 p.o. instillation (6 months)	10^10.0^ CCID50	60 days	0/5		0/5	0/5	5/5	[35]
	5 p.o. bait (6 months)	10^9.9^ CCID50	60 days	0/5		0/5	0/5	5/5	

p.o. = per os, D = day, p.f.u. = plaque forming units, CCID50 = cell culture infective dose 50%.

Seronegative foxes received by direct instillation into the mouth ten times (10^9.0^ PFU/animal) or more than 60 times the recommended dose (10^9.8^ CCID_50_/animal) of SAG2 vaccine [26]. All foxes remained in good health during the 6-month observation period. No virus excretion was detected in any saliva samples collected 1, 3 and 6-7 days after vaccination. All brain samples (hippocampus, medulla oblongata, cortex) and salivary glands from vaccinated foxes were negative for rabies virus antigen detection (by the direct immunofluorescence test) or for rabies virus isolation, by inoculation in neuroblastoma cells [26].

The safety of a repeated administration of a field dose was also evaluated in the fox. Fifteen seronegative foxes consumed one (*n* = 5 animals), two (*n* = 5) or three (*n* = 5) baits on 3 consecutive days [26]. The foxes remained healthy during the 32-day observation period. No rabies virus was detected in any brain, salivary glands or tonsils by the direct immunofluorescence test and cell culture in any animal [26]. Thus, in the event of the ingestion of several baits in the field, no harmful effects were expected.

The spread of the SAG2 vaccine strain was also studied. The SAG2 strain is a modified live-rabies virus, and as are most enveloped viruses, the virus is sensitive to inactivation by acids and bases, ultraviolet light, heat, repeated freeze-thaw cycles and disinfectants such as mild soap solutions [33]. Thus the SAG2 virus is likely to be inactivated in the event of release in the environment. Transmission from animal to animal could only occur through saliva, and not through faeces, because the vaccine virus is inactivated rapidly in the stomach [34]. In consequence, the spreading of the SAG2 strain was studied through saliva swabs and in salivary glands. No sample was detected positive for rabies virus in any fox after the administration of high or repeated doses of the SAG2 strain (see previous paragraph).

The safety profile observed in the fox was confirmed in the raccoon dog. A SAG2 suspension was instilled in the mouth of five raccoon dogs (10^10.0^ CCID_50_/animal), and each of five other raccoon dogs consumed a SAG2 bait (10^9.9^ CCID_50_/bait) [35]. All raccoon dogs remained healthy during the 60-day observation period. Brains and salivary glands from all animals were negative for rabies virus. The safety of the oral administration of SAG2 vaccine was demonstrated in two species of jackal (side-striped jackal and black-backed jackal) either by direct instillation (the highest dose was 10^7.5^ CCID_50_/animal) or included in a bait (the highest dose was 10^8.0^ CCID_50_/animal) [30]. In addition, all saliva swabs collected 1, 3 and 7 days after vaccine instillation were negative for rabies virus detection except for a pool of saliva swabs collected on day 3 from three black-backed jackals inoculated with the highest dose (10^7.5^ TCID_50_/animal) of the vaccine [30].

### 3.2 Non target species

Any animal species sharing the habitat of the target species may ingest vaccine baits. The most relevant and common non-target species were selected in different countries and inoculated with the SAG2 strain (Table 2). The SAG2 studies were conducted according to the guidelines of WHO for the safety testing of modified live-rabies vaccines for use in wild carnivores [4] and according to the European Pharmacopoeia [32]. For some trials performed in countries where the SAG2 vaccine was intended to be used in dogs, recommendations given by WHO for safety testing of oral vaccines for domestic dogs were applied (wherever possible) because they are more stringent, dogs being in contact with humans [5,6]. In particular, modified live-virus vaccines for domestic dogs must be safe for local wild and domestic non-target animal species when administered orally at 10 times the recommended dose used in the field, and there should be no, or minimal, excretion of virus following vaccination and there should be no persistent infection by the vaccine virus. In addition, vaccines should be assessed and found safe in non-human primates.

**Table 2:** **Summary of safety studies in various target and non target species**

**Order**	**Family**	**Species**	**No of animals (route of administration)**	**SAG dose per animal (volume)**	**Time of observation**	**Source**
*Rodentia*	*Cricetidae*	Common vole	9 p.o.	10^7.7^ PFU (0.05 mL)	78 days	[36]
		*(Microtus arvalisà*)	9 i.m.	10^7.5^ PFU (0.03 mL)	78 days	
*Rodentia*	*Cricetidae*	Bank vole (*Myodes glareolus*)	11 p.o.	10^7.7^ PFU (0.05 mL)	78 days	[36]
			11 i.m.	10^7.5^ PFU (0.03 mL)	78 days	
*Rodentia*	*Cricetidae*	European water vole	2 p.o.	10^7.7^ PFU (0.05 mL)	78 days	[36]
		(*Arvicola amphibius*)				
*Rodentia*	*Cricetidae*	Tundra vole	9 p.o.	10^7.16^ MICLD_50_ (0.03 mL)	46 days	[40]
		(*Microtus oeconomus*)				
*Rodentia*	*Cricetidae*	Northern red-backed vole *(Myodes rutilus)*	10 p.o.	10^7.16^ MICLD_50_ (0.03 mL)	46 days	[40]
*Rodentia*	*Sciuridae*	Arctic ground squirrel	9 p.o.	10^7.68^ MICLD_50_ (0.1 mL)	46 days	[40]
		(*Spermophilus parryii*)				
*Rodentia*	*Muridae*	Field mouse	15 p.o.	10^7.5^ PFU (0.05 mL)	78 days	[36]
		(*Apodemus flavicollis*	15 p.o.	10^7.7^ PFU (0.03 mL)	78 days	
		or *Apodemus sylvaticus*)	10 i.c.	10^7.5^ PFU (0.03 mL)	78 days	
*Rodentia*	*Muridae*	Norway rat	15 p.o.	10^7.44^ PFU (0.05 mL)	42 days	[36]
		(*Rattus norvegicus*)	15 i.m.	10^7.44^ PFU (0.05 mL)	42 days	
*Rodentia*	*Muridae*	Multi-mammate mouse	39 p.o.	10^8.0^ TCID_50_ (0.1 mL)	≥ 90 days	[37]
		(*Mastomys natalensis*)				
*Rodentia*	*Muridae*	Bushfelt gerbil	26 p.o.	10^8.0^ TCID_50_ (0.1 mL)	≥ 90 days	[37]
		(*Gerbilliscus leucogaster*)				
*Rodentia*	*Muridae*	North African gerbil *(Gerbillus campestris)*	32 i.m.	10^7.0^ - 10^8.5^ TCID_50_ (0.03-0.1 mL)	60 days	[38]
*Rodentia*	*Muridae*	Merion	14 p.o.	10^8.2^ - 10^9.2^ TCID_50_ (0.05-0.5 mL)	60 days	[38]
		*(Meriones)*	14 i.m.	10^7.0^ - 10^8.5^ TCID_50_ (0.03-0.1 mL)	60 days	
*Rodentia*	*Dipodidae*	Greater Egyptian Jerboa *(Jaculus orientalis)*	2 i.m.	10^7.0^ TCID_50_ (0.03 mL)	60 days	[38]
*Carnivora*	*Canidae*	Red fox (*Vulpes vulpes*)	10 p.o.	10^9.8^ TCID_50_ (2 mL)	183 days	[26]
*Carnivora*	*Canidae*	Black-backed jackal	3 p.o.	10^7.5^ TCID_50_ (1 mL)	180 days	[30]
		(*Canis mesomelas*)	3 p.o.	10^6.5^ TCID_50_ (1 mL)	180 days	
*Carnivora*	*Canidae*	Side-striped jackal	3 p.o.	10^7.5^ TCID_50_ (1 mL)	180 days	[30]
		(*Canis adustus*)	3 p.o.	10^6.5^ TCID_50_ (1 mL)	180 days	
*Carnivora*	*Canidae*	Golden jackal (*Canis aureus*)	8 p.o.	10^9.5^ TCID_50_ (1 mL)	90 days	[38]
*Carnivora*	*Canidae*	Western coyote (*Canis latrans*)	5 p.o. 4 p.o.	10^8.3^ TCID_50_ (bait) 10^9.6^ TCID_50_ (bait)	30 days 30 days	(Rupprecht et al.: Efficacy of SAG2 for oral vaccination of coyotes against rabies, unpublished)
*Carnivora*	*Canidae*	Domestic dog (*Canis familiaris*)	21 p.o.	10^9.5^ TCID_50_ (1 mL)	90 or 180 days	[38]
*Carnivora*	*Canidae*	Wild dog *(Lycaon pictus)*	4 p.o.	10^8.0^ TCID_50_ (1 mL)	634 days	[39]
*Carnivora*	*Canidae*	Raccoon dog	5 p.o.	10^10.0^ CCID_50_ (1.7 mL)	60 days	[35]
		*(Nyctereutes procyonoides)*	5 p.o.	10^9.8^ CCID_50_ (bait)		
*Carnivora*	*Felidae*	Domestic cat (*Felis catus*)	10 p.o.	10^10^ TCID_50_ (1 mL)	180 days	[26]
*Carnivora*	*Felidae*	Domestic cat (*Felis catus*)	11 p.o.	10^9.5^ TCID_50_ (1 mL)	90 days	[38]
*Carnivora*	*Mustelidae*	Domestic ferret	4 p.o.	10^8.17^ PFU (2 mL)	37 days	[36]
		(*Mustela putorius furo*)	4 i.m.			
*Carnivora*	*Mustelidae*	Honey badger (*Mellivora capensis*)	6 p.o.	10^9.0^ TCID_50_ (1 mL)	≥ 90 days	[37]
*Carnivora*	*Mustelidae*	European badger (*Meles meles*)	5 p.o.	10^8.17^ PFU (2 mL)	35 days	[36]
*Carnivora*	*Viverridae*	African civet	6 p.o.	10^9.0^ TCID_50_ (1 mL)	≥ 90 days	[37]
		(*Civettictis civetta*)				
*Carnivora*	*Viverridae*	Large-spotted genet	6 p.o.	10^9.7^ TCID_50_ (1 mL)	≥ 90 days	[37]
		(*Genetta tigrina*)				
*Carnivora*	*Herpestidae*	Slender mongoose	6 p.o.	10^9.0^ TCID_50_ (1 mL)	≥ 90 days	[37]
		(*Galerella sanguinea*)				
*Carnivora*	*Procyonidae*	Raccoon *(Procyon lotor)*	5 p.o.	10^9.0^ TCID_50_ (1 mL)	30 days	[55]
*Carnivora*	*Mephitidae*	Striped skunk *(Mephitis mephitis)*	5 p.o.	10^9.0^ TCID_50_ (1 mL)	30 days	[55]
*Primates*	*Cercopithecidae*	Chacma baboon (*Papio ursinus*)	10 p.o.	10^9.0^ TCID_50_ (1 mL)	≥ 90 days	[37]
*Erinaceomorpha*	*Erinaceidae*	Western European hedgehog (*Erinaceus europaeus*)	6 p.o.	10^7.87^ PFU (1 mL)	57 days	[36]
*Artiodactyla*	*Suidae*	Wild boar (*Sus scrofa*)	5 p.o.	10^8.88^ PFU (2 mL)	35 days	[36]
*Artiodactyla*	*Bovidae*	Domestic goat (*Capra hircus*)	6 p.o.	10^8.8^ PFU (2 mL)	35 days	[36]
*Artiodactyla*	*Bovidae*	Cow *(Bos primigenius)*	5 p.o.	10^10.0^ CCID_50_	60 days	[26]
*Passeriformes*	*Corvidae*	Carrion crow (*Corvus corone*)	7 p.o.	10^8.66^ PFU (1.5 mL)	33 days	[36]
*Passeriformes*	*Corvidae*	Pied crow (*Corvus albus*)	6 p.o.	10^9.0^ TCID_50_ (1 mL)	≥ 90 days	[37]
*Passeriformes*	*Corvidae*	Rook (*Corvus frugilegus*)	8 p.o.	10^8.66^ PFU (1.5 mL)	33 days	[36]
*Falconiformes*	*Accipitridae*	Buzzard (*Buteo buteo*)	7 p.o.	10^8.18^ PFU (1.0 mL)	33 days	[36]
*Falconiformes*	*Accipitridae*	Red kite (*Milvus milvus*)	1 p.o.	10^8.18^ PFU (1.0 mL)	33 days	[36]
*Strigiformes*	*Strigidae*	Tawny owl (*Strix aluco*)	1 p.o.	10^8.18^ PFU (1.0 mL)	33 days	[36]
*Strigiformes*	*Strigidae*	Long-eared owl (*Asio otus*)	2 p.o.	10^8.18^ PFU (1.0 mL)	33 days	[36]
*Strigiformes*	*Tytonidae*	Barn owl (*Tyto alba*)	1 p.o.	10^8.18^ PFU (1.0 mL)	33 days	[36]

TCID_50_: median tissue culture infectious doses.

p.f.u.: plaque forming units.

MICLD_50_: mouse intracerebral lethal dose (10^7.2^ MICLD_50_ equivalent to 10^9.0^ CCID_50_).

p.o. = per os, i.m. = intramuscularly.

The safety of the SAG2 strain was demonstrated in various European [36], African [37–39] and Arctic [40] non-target species including rodents (mouse, rat, vole, squirrel, gerbil, jerboa, meriones), carnivores (coyote, ferret, civet, mongoose, badger, genet), non-human primates (Chacma baboons), other mammals (hedgehog, wild boar, domestic goat, cow), and diurnal and nocturnal birds (crow, rook, buzzard, kite, owl). All species received the SAG2 vaccine by direct instillation into the mouth, and for some species by intramuscular or intracerebral route. None of the inoculated animals showed specific lesions or died of rabies during the observation period. Importantly, no pathogenicity of the SAG2 strain administered orally at a high dose (10^9^ TCID_50_/animal) was observed in the baboon [37].

Different studies in non target species showed that the persistence of the SAG2 virus in the oral cavity is very limited or even nil in non target species. In a study performed in Zimbabwe [37], saliva swabs were collected from all animals (except rodents and crows), 1, 3 and 7 days after vaccine administration. Only one saliva sample was detected positive (at a very low virus concentration) in one genet 1 day after vaccine administration [37]. The presence of a residual vaccine inoculum 24 h after oral administration of the SAG2 strain to the genet could not be excluded. No persistent infection after administration of the SAG2 strain was detected in any of the brain and salivary glands (or saliva) from civets, or for some animals in tonsil tissues at the end of the observation period [36,37].

In most studies, at least two WHO/OIE methods were used for the detection of rabies virus, i.e. the direct fluorescent antibody test (FAT) [41] on smears of brain or salivary glands, and the inoculation of brain or salivary glands homogenates to cell culture [42] or intracerebrally to 1-3 day old mice [43].

The safety of a lyophilized form of the SAG2 vaccine included in a bait was also demonstrated in the northern collared lemming, *Dicrostonyx groenlandicus* [44].

## 4. Immunogenicity and efficacy in wildlife animals in controlled laboratory trials

Although the best approach for testing a vaccine is to evaluate it in the field, it is necessary to first determine efficacy under controlled conditions.

SamplesBlood samples from foxes and raccoon dogs were collected in the field and in ANSES-Nancy’ experimental station (Atton, Meurthe-et-Moselle département, France). The facility has been approved by French Veterinary Services on 19 of April 2011 (approval C-54-431-1). Experiments and husbandry were conducted following European Directive, 2010/63/EU and French regulations on ethics in animal experimentation.

The immunogenicity of the SAG2 strain was then assessed in different wild animal species (Table 3).

**Table 3:** **Summary of the efficacy data in different target species after vaccination with SAG2 vaccine administered either by direct instillation in the mouth or by bait consumption**

**Species**	**Mean age**	**No of animals, route**	**SAG dose per animal**	**Challenge after vaccination**	**Seroconversion**	**Survival after challenge**	**Rabies virus positive brains**	**Source**
*Red fox*	± 12 months	5 p.o.	10^7^ p.f.u.	29 days	5/5	5/5	0/5	[31]
	± 12 months	5 p.o.	10^8^ p.f.u.		5/5	5/5	0/5	
	± 12 months	5 controls	-		0/5	0/5	5/5	
*Red fox*	± 9 months	6 p.o.	10^7.5^ p.f.u.	85 days	6/6	6/6	0/6	[26]
	± 9 months	2 controls	-		0/2	0/2	2/2	
	± 9 months	5 p.o.	10^7.5^ p.f.u.	220 days	5/5	5/5	0/5	
	± 9 months	1 control	-		0/1	0/1	1/1	
	± 9 months	6 p.o.	10^7.5^ p.f.u.	303 days	6/6	6/6	0/6	
	± 9 months	2 controls	-		0/2	0/2		
*Red fox*	13-14 months	5 p.o. (bait)	10^7.0^ CCID_50_	30 days	5/5	4/5	1/5	[26]
		5 p.o. (bait)	10^8.0^ CCID_50_		5/5	4/5	1/5	
		5 p.o. (bait)	10^9.0^ CCID_50_		5/5	5/5	0/5	
		2 controls	-		0/2	0/2	2/2	
*Red fox*	6.4 months to 4.5 years	25 p.o. (bait)	10^8.0^ CCID_50_	180 days	21/25	22/25	3/25	[26]
		10 controls	-		0/10	0/10	10/10	
*Raccoon dog*	6 months	29 p.o. (23 bait, 6 instill)	10^8.15^ CCID_50_	201 days	29/29	28/28	0/28	[35]
	6 months	12 controls	-		0/12	0/11	11/11	
*Side-striped jackal*	≥ 9 months	3 p.o. (instill)	10^6.5^ CCID_50_	6 months	2/3	3/3	0/3	[30]
	≥ 9 months	3 p.o. (instill)	10^7.5^ CCID_50_		2/3	2/3	1/3	
	≥ 9 months	3 controls	-		0/3	0/3	3/3	
*Black-backed jackal*	≥ 9 months	3 p.o. (instill)	10^6.5^ CCID_50_	6 months	3/3	3/3	0/3	[30]
	≥ 9 months	3 p.o. (instill)	10^7.5^ CCID_50_		3/3	3/3	0/3	
	≥ 9 months	2 controls	-		0/2	0/2	2/2	
*Side-striped jackal*	≥ 9 months	5 p.o. (bait)	10^8.25^ CCID_50_	30 days	5/5	5/5		[30]
	≥ 9 months	5 p.o. (bait)	10^7.25^ CCID_50_		3/5	3/5		
	≥ 9 months	5 controls			0/5	0/5		
*Golden jackals*	≥ 9 months	14 p.o. (bait)	10^8.0^ CCID_50_	160 days	11/14	12/14 (85.7%)		[50]
	≥ 9 months	10 controls	-		0/10	0/10 (0%)		
*Arctic fox*		10 p.o. (bait)	10^7.75^ SMICLD_50_	7 weeks	10/10	10/10	0/10	[52]
		4 controls	-		0/4	1/4	3/4	
*Raccoon*	adult	5 p.o. (instill)	10^9.0^ TCID_50_	30 days	3/5	5/5	0/5	[55]
	adult	5 controls	-		0/5	0/5	5/5	
*Striped skunk*	adult	5 p.o. (instill)	10^9.0^ TCID_50_	30 days	2/5	5/5	0/5	[55]
	adult	5 controls	-		0/5	0/4*	4/5	
*Coyotes*	unknown	4 p.o. (liquid bait form)	10^7.3^ TCID_50_	30 days	2/4	3/4	1/4	(Rupprecht et al.: Efficacy of SAG2 for oral vaccination of coyotes against rabies, unpublished)
	unknown	4 p.o. (liquid bait form)	10^8.3^ TCID_50_		3/4	4/4	0/4	
	unknown	4 p.o. (liquid bait form)	10^9.6^ TCID_50_		4/4	4/4	0/4	
	unknown	5 p.o. (lyophilized bait form)	10^6.9^ TCID_50_		3/5	2/5	3/5	
	unknown	5 p.o. (lyophilized bait form)	10^7.2^ TCID_50_		3/5	4/5	1/5	
	unknown	5 p.o. (lyophilized bait form)	10^8.3^ TCID_50_		5/5	5/5	0/5	
	unknown	5 controls			0/5	0/5	5/5	

*1 control skunk died following sedation on day 83 and was negative for rabies.

p.o. = per os, p.f.u. = plaque forming units, CCID_50_ = cell culture infective dose 50%, TCID_50_ = median tissue culture infectious dose 50%, SMICLD_50_ = suckling mouse intracranial 50% lethal dose.

Groups of ten 6-week old mice were inoculated intracerebrally with 30 μL of various dilutions of the SAG2 strain (equivalent to 1.5 – 2.5 – 3.5 – 4.5 – 5.5 and 6.5 log PFU/mouse). Four weeks later, mice were challenged with 200 LD_50_ of the CVS strain of rabies virus in the masseter muscle. From a dose of 3.5 log PFU/mouse, all mice were protected [17].

### 4.1 Red fox

Red foxes (*Vulpes vulpes*) are responsible for the maintenance and spread of rabies in the subarctic and north-eastern parts of North America, in subarctic Asia, and in central and eastern Europe.

Two experiments [26,31] demonstrated that the SAG2 strain administrated by instillation into the mouth at different doses (10^8^ (*n* = 5), 10^7.5^ (*n* = 18), 10^7^ (*n* = 5) PFU/animal) induced a full protective immunity after a rabies virus challenge performed 29, 85, 220 or 303 days after oral instillation (Table 3). In a third experiment [26], different doses of the SAG2 vaccine (10^7.0^, 10^8.0^ and 10^9.0^ CCID_50_/bait, *n* = 5 animals/dose) included in a bait were administered to foxes. Baits were consumed within 1 hour by 13 of 15 foxes, showing the attractiveness of the bait. All vaccinates seroconverted, a dose-response relationship was observed between the mean neutralizing antibody titers and the dose of vaccine (1.7, 2.5 and 5.1 IU/mL in the 10^7.0^, 10^8.0^ and 10^9.0^ CCID_50_ dosage groups, respectively). All controls succumbed to rabies virus challenge performed 30 days after vaccination, whereas high survival rates (80%, 80% and 100% in the 10^7.0^, 10^8.0^ and 10^9.0^ CCID_50_ dosage groups) were observed in challenged vaccinates (Table 3). This study showed that the minimal protective dose is probably below the lowest dose tested.

The last study [26] was conducted according to the requirements of the European monograph (0746) [32]. Twenty-five foxes received a SAG2 bait at the recommended dosage (10^8^ CCID_50_/bait). All animals (25 vaccinates and 10 controls) were challenged 180 days after vaccination. All controls and three of 25 vaccinates developed clinical signs and died of rabies (Table 3). Two of three not protected foxes did not eat the bait. No seroconversion was observed in the three vaccinates which were not protected, while all survivors except one vaccinate seroconverted after vaccination.

These different trials demonstrated the protective effect of SAG2 vaccine bait in controlled laboratory conditions. Obviously, the puncture of the capsule is a determinant factor for ensuring the protection.

Although seroconversion is usually associated with immunity against a rabies virus challenge, in the various experiments some animals did not seroconvert and were protected after challenge. A study showed that a T-cell specific memory response was detected in foxes 6 months after the oral instillation of a field dose of SAG2, suggesting that the SAG2 vaccine can stimulate cell-mediated mechanisms in foxes [45].

A long-lasting protective immunity was demonstrated in red foxes immunized with the SAG1 strain administered by instillation in the oral cavity (10^7^ TCID_50_/dose) up to 18 months after vaccination [46]. Since SAG1 and SAG2 strains have a comparable efficacy profile, it may be hypothesized that the SAG2 strain is also able to induce a long-term protection of red foxes lasting for the average life of a fox. However, a bi-annual vaccination is required in the field, in particular to target juveniles during spring [29].

### 4.2 Raccoon dog

The raccoon dog (*Nyctereutes procyonoides*), a member of the Canidae, has been introduced by the fur industry from eastern Asia into the European part of Russia around 1920. This species has spread rapidly in Europe due to its high rate of reproduction, extreme adaptability, omnivorous habits and capacity to hibernate [47]. Raccoon dogs are now common in the Baltic States, many parts of eastern Europe and Finland with a density even higher than that of the red fox. Its presence has been reported in Germany, Sweden, Norway, France, The Netherlands, Switzerland, Poland and Austria [47]. The raccoon dog has become a major factor in the epidemiology of rabies in the Eastern and Northern Europe [47,48] and is now the second most important wildlife species – after the red fox – infected with rabies [49]. Faced with the increase of rabies cases in raccoon dogs, specific investigations to assess the efficacy of OV were conducted under controlled conditions (Table 3). SAG2 baits (one bait/animal) at a field dose (10^8.15^ CCID_50_/dose) were given to 29 raccoon dogs [35]. Baits were consumed rapidly by most animals (the other animals were instilled in the oral cavity with the content of the bait). All vaccinated raccoon dogs developed high neutralizing antibody titers. All vaccinates (*n* = 29) were fully protected against a rabies virus challenge performed 201 days after vaccination, while all controls (*n* = 12) succumbed to rabies [35]. SAG2 vaccine baits are attractive for raccoon dogs and induce a protective immunity against rabies.

### 4.3 Jackal

There are three species of jackals in Africa and Asia. In Africa, jackals are one of the major wildlife rabies vectors [27]. In Zimbabwe, a quarter of rabies cases are reported in jackals, especially in the side-striped (80% of jackal cases) and black-backed jackal [30].

The efficacy of the SAG2 vaccine was assessed in these two species [30]. SAG2 was administered either by direct oral instillation at two doses (10^6.5^ or 10^7.5^ CCID_50_/animal, *n* = 3 animals/dose) (Table 3) or placed into blisters stapled under the skin of chicken heads at two doses (10^7.25^ or 10^8.25^ CCID_50_/animal, *n* = 5 animals/dose). All animals were challenged either 1 month (vaccinated with a bait) or 6 months (vaccinated by instillation) after vaccination using a rabies virus field strain of side-striped jackal (salivary glands). Most jackals seroconverted after vaccination and high survival rates were observed after direct oral administration in both species [30].

These promising results were confirmed in the golden jackal, *Canis aureus* (Table 3). The golden jackal is found from Yugoslavia and Greece to Burma in Asia, including Turkey and India, the Middle East and southern parts of the Arabian Peninsula [50]. Between 1950 and 1970, the golden jackal represented a major reservoir of wildlife in Israel [50]. Golden jackals received SAG2 baits and were challenged 160 days after vaccination with a local rabies virus isolate from a jackal [50]. Vaccinated animals achieved a seroconversion rate of 79% 150 days after vaccination, and high protection rate after challenge was observed (86%). In contrast all controls succumbed to rabies [50].

### 4.4 Arctic fox

The arctic fox (*Alopex lagopus*) is the primary rabies reservoir in most circumpolar regions [51]. Since freezing temperatures are observed throughout the year in arctic regions, the liquid form of the SAG2 vaccine was not adapted, because it could freeze and passed directly into the stomach without a step of absorption through the oral mucosa. To assess the efficacy of the SAG2 vaccine, the SAG2 vaccine was lyophilized in the form of a wafer coated by a thin layer of ground beef. Ten arctic foxes received a prototype SAG2 bait, at 10^7.75^ SMICLD_50_ [52]. All baits were consumed rapidly. Oral swabs collected 1, 24, 48 and 72 h after bait intake were all negative for rabies virus. All vaccinates seroconverted by 2 weeks after vaccination and had neutralising antibody titers (≥ 0.5 IU/mL) just before challenge. All vaccinates and four controls were challenged 7 weeks after vaccination with an arctic red fox rabies virus strain (isolated in Alaska). All vaccinates survived rabies virus challenge whereas three of four controls died of rabies [52].

### 4.5 Raccoon and skunk

The raccoon (*Procyon lotor*) and striped skunk (*Mephitis mephitis*) are major wildlife rabies reservoirs in North America [53,54]. Although SAG2 is not licensed in North America, its utility for local wildlife OV was assessed. Five raccoons and five skunks received by instillation in the mouth a suspension of SAG2 (10^9^ TCID_50_/animal) [55] (Table 3). Three of five raccoons and two of five skunks seroconverted after vaccination. All animals were challenged 30 days after vaccination with either a raccoon rabies virus pool from salivary glands or a skunk rabies virus isolate. All vaccinated animals resisted challenge while all control raccoons (*n* = 5) and all control skunks (4 out of 4, the 5^th^ control died following sedation and was negative for rabies virus) died of rabies.

### 4.6 Mongoose

In South Africa, the yellow mongoose is the main reservoir of rabies [56]. The immunogenicity of the SAG2 vaccine was evaluated in mongooses vaccinated by oral instillation: 13 of 15 vaccinated mongooses were protected after a virulent rabies virus challenge [6].

### 4.7 Coyote

A dose response experiment was conducted to assess the efficacy of SAG2 baits in coyotes (Rupprecht et al.: Efficacy of SAG2 for oral vaccination of coyotes against rabies, unpublished). Different doses of the liquid form (10^7.3^, 10^8.3^, 10^9.6^ TCID_50_/bait, *n* = 4 coyotes per dose) or lyophilized bait form (10^6.9^, 10^7.2^, 10^8.3^ TCID_50_/bait, *n* = 5 coyotes per dose) of SAG2 vaccine were administered (Table 3). Five coyotes served as controls. High seroconversion rates were observed. On day 30, all coyotes were challenged with a coyote street rabies virus. All 5 controls succumbed to challenge, as did only one of 12 animals from the group vaccinated with the liquid bait form (at the lowest dilution) and 4 of 15 from the group vaccinated with the lyophilized bait form (3 at the lowest dilution, and 1 at the next concentration, but none at the highest concentration). Thus, SAG2 vaccine used at a field concentration of 10^8.3^ TCID_50_ could be used for the protection of coyotes against rabies (Rupprecht et al.: Efficacy of SAG2 for oral vaccination of coyotes against rabies, unpublished).

### 4.8 Ethiopian wolf

The Ethiopian wolf (*Canis simensis*) is highly endangered and the rarest canid in the world, with only about 420 adults living in the Ethiopian highlands [57]. Rabies outbreaks occurred during 1991, 2003 and 2008/2009 with a high mortality rate of up to 75% of the affected population [57]. Emergency parenteral vaccination of several packs of Ethiopian wolves was initiated during 2003 and 2008-09 to contain outbreaks [57]. However, parenteral vaccination is logistically complex and costly. Thus, during 2011 another approach was considered [57,58]. A small pilot study was conducted to assess the efficacy of SAG2 vaccine. The animals received a SAG2 vaccine blister inserted into goat/sheep meat. Among the four animals which consumed bait, three seroconverted [57]. All these wolves were still alive and healthy almost one year after vaccine administration (Chris Gordon, personal communication).

Other studies conducted to evaluate the safety of the SAG2 strain in different species showed that after oral administration, rabies virus neutralizing antibodies were produced in the ferret, badger, wild boar, mouse, meriones, civet, mongoose, jackal, wild dogs (*Lycaon pictus*), captive-bred baboon, and raptors, but none in the goat, Norway rat, vole or Corvidae [36–39].

Thus, the efficacy of SAG2 baits was demonstrated according to the EU requirements for the fox and raccoon dog. Encouraging results were obtained in the jackal, raccoon, skunk, mongoose and Ethiopian wolf.

## 5. Efficacy of SAG2 in the field

The SAG2 vaccine (RABIGEN®, Virbac Laboratories, Carros, France) has been registered in the 28 countries of the EU (European Medicines Agency registration) for OV administration in baits to foxes and raccoon dogs [26].

The SAG2 vaccine has contributed substantially to the elimination of rabies in several European countries. The efficacy of OV is assessed by comparing the prevalence of rabies in vaccinated areas, the proportion of fox and raccoon dog populations which consume the bait (revealed through a tetracycline biomarker) and immunisation rates by antibody measurement [27,29].

The number of campaigns and associated costs necessary to reach the rabies free status depended on various parameters mainly linked to the planning and the implementation of OV, the rabies situation and control programmes in neighbouring countries and the level of surveillance. The strategies of oral vaccination using SAG2 vaccine were different depending on the countries (as exposed in the following paragraphs).

### 5.1 Switzerland

**Figure 3:**
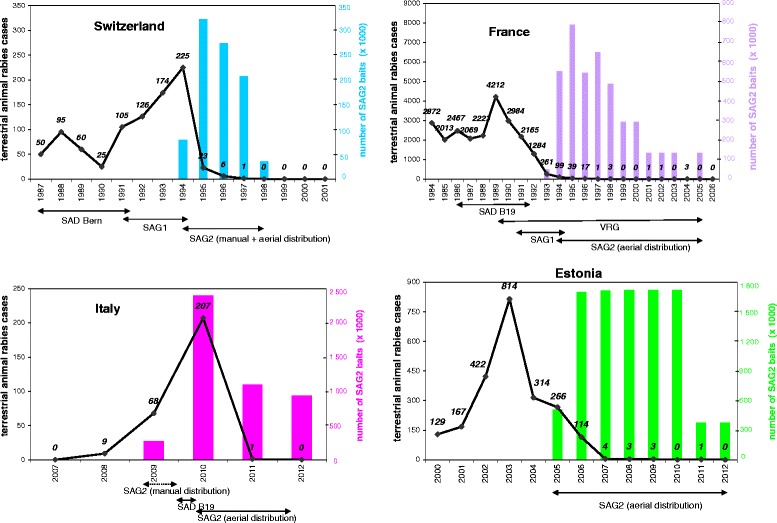
**Animal rabies cases before and after vaccination campaigns with SAG2 in Switzerland, France, Estonia, Italy.** The impact of the vaccination with SAG2 baits on the number of terrestrial animal rabies cases is illustrated in four countries, Switzerland, France, Estonia and Italy.

The fox rabies epidemic, originating from eastern Europe, reached Switzerland during 1967 [59]. Initial attempts to stop disease progression consisted in reducing fox population (by gassing in fox dens and shooting foxes at the den). This strategy did not succeed in preventing outbreaks and rabies spread almost throughout the territory. A peak in rabies cases was observed during 1976 (1738 cases) with 73% of cases reported in foxes [59]. Switzerland was the first European country to initiate a limited field trial of OV of foxes during October 1978 in the lower Rhône Valley (canton of Valais). The disease did not cross the resulting barrier (approximately 60% of foxes were believed to be immune). The OV campaigns were extended gradually. Capsules of SAD Bern vaccine included in chicken head baits were distributed manually during spring and autumn [59,60]. The country was divided into epidemiologic compartments delineated by natural (such as high mountain ranges, rivers, lakes) and artificial obstacles to the spread of rabies. The strategy adopted for OV was to treat infected compartments one by one until they were free of rabies, to protect the entrances to rabies-free by threatened compartments [60]. The number of rabies cases decreased to 25 during 1990 (Figure 3), and a major part of the country was freed from rabies, except for one area in the Jura mountains close to the French border [61–63]. A new outbreak of rabies occurred during 1991 in the Jura, probably originating from France. During spring 1991, SAD Bern chicken head baits were replaced by industrially manufactured fishmeal/beef SAG1 baits. SAG1 and SAG2 baits were used during 1994, and by 1995 SAG2 replaced SAG1 baits (Figure 3). Rabies cases remained high (225 cases during 1994), so the vaccination strategy was adjusted. The bait density was increased from 15 to 25 baits per km^2^ to take into account a larger fox population and density, two vaccination campaigns were conducted within 1 month in any new vaccination area and baits were distributed at fox den entrance (6 to 10 baits/fox den) in an additional campaign during early summer to immunize young foxes [61–63]. Cross-border vaccination efforts limited the risk of reinfection from neighbouring countries. Vaccination was also continued until at least 2 years after recording the last case of rabies related to the fox rabies epidemic. As a consequence, the number of rabies cases dropped from 23 to 6 during 1995 and 1996, respectively [61–63]. Since 1997, i.e. less than 2 years after initiation of this new strategy, no indigenous rabies cases were reported. Switzerland was declared rabies-free during spring 1999 [61–63].

Comparing bait uptake and seroconversion rates, during 1995 and 1996, 66% of foxes tested were detected as positive for tetracycline and 50% had rabies virus neutralizing antibodies [63]. The proportion of foxes positive for tetracycline and neutralizing antibodies was higher in adults (75% and 59%, respectively) than young foxes (58% and 40%, respectively). Seasonal variations were observed in young foxes (< 1 year of age) with a higher bait uptake and seroconversion rate after the autumn campaigns than the spring. Interestingly, the den baiting strategy led to a significant increase in tetracycline and seroconversion rates in young foxes (64% and 50%, respectively), reaching values close to that observed in adults [63].

### 5.2 France

Fox rabies invaded north-eastern France during 1968. The first case was reported in Moselle, near the German border [64]. From 1968 to 1974, rabies progressed at a mean annual speed of 30-40 km, and then the infectious wave front slowed [62]. During 1981, old foci were reactivated and rabies increased. From 1986 to 1988, the first OV campaigns were conducted in the Lorraine region and the French Jura and Alps in collaboration with Belgium, Luxembourg and Switzerland [18]. Manual baiting was used for the first campaigns, and after 1987, aerial methods were adopted for the distribution of larger quantities of baits. Since autumn 1988, helicopters flying at an altitude of 60-100 m and a speed of 180 km/h along three transect lines per km^2^ were preferred over lighter aircrafts [64]. Despite encouraging results, rabies progressed and crossed the Seine and the Loire rivers. During 1989, the infected area covered 140 000 km^2^, corresponding to the highest case numbers observed in France (4212 terrestrial rabies cases reported) (Figure 3). To protect south-western France, which was still rabies-free, rather than vaccinating rabies from the north of Lorraine (surrounded by heavily infected areas), a continuous strip (50 km wide) was established during autumn 1990 from the English Channel to the Swiss border [62]. Rabies OV was extended to the north and the east towards the borders with Belgium, Luxembourg and Germany [62]. The OV campaigns were performed during autumn and spring in the entire contaminated area (141 700 km^2^) from autumn 1992 to 1997 [18]. The number of rabies cases decreased rapidly. From 1997 to 2000, OV campaigns were conducted along the borders with Switzerland and with Germany [62]. The last three cases of rabies (two foxes and one cat) were registered in Moselle near the German border between November 1997 and December 1998. The cases reported in Figure 3 in 2001, 2002 and 2004 were rabid dogs imported from countries infected with canine rabies. From 2001 to 2003 and in 2005 (last campaign in autumn), OV was limited to a border area of 5500 km^2^ with a bait density of 20 baits/km^2^ [18,65]. France has been recognized as free of terrestrial cases since 2001 [18,62,64]. Since the fox populations were growing, the bait density was increased from 13 baits/km^2^ during 1986 to 1995, to 15 baits/km^2^ during 1995 and 20 baits/km^2^ since 1997 [65]. Four different vaccines were used between 1986 and 1998: SAD B19 was used from 1986 to 1991, and was gradually replaced by V-RG, during autumn 1989 and SAG1 from autumn 1990 [64]. The SAG2 vaccine was used for the first time in France during autumn 1993. Thereafter, SAG1 was replaced by SAG2 during spring 1994. More than 3 million SAG2 baits were distributed from autumn 1993 to 1998. Overall, during the 1994-1997 period, 74% of foxes (84% of adults and 60% of fox cubs) were found positive for tetracycline [26]. The proportion of foxes (all age groups combined) found positive for tetracycline increased gradually from 50% and 66% after the spring and autumn campaigns during 1994, respectively to 87% and 90% for the corresponding seasons during 1997. As for Switzerland, the proportion of foxes found positive for tetracycline was lower after the spring campaign than after the autumn during 1995 and 1996 [26]. The increase in the positivity rate for tetracycline during the 1991-1997 period was more marked for young foxes (during spring and autumn, with 35% and 37% during 1994, 81% and 84% during 1997, respectively) that adult foxes (in spring and autumn, with 89% and 74% during 1994, 93% and 92% during 1997, respectively) [26]. The analysis of fox teeth showed several tetracycline lines, indicating that multiple baits were consumed by individual foxes. On average, 2.6 baits were consumed by adult foxes and 2.1 baits by fox cubs during the 1994-1997 periods [66]. During this period, 64% of foxes found positive for tetracycline (71% for adults and 51% for fox cubs) were seropositive for rabies virus antibodies using an ELISA test [26].

Different strategies have been evaluated in France to improve the efficacy of OV in the field, such as increasing the number of vaccine baits distributed during one campaign, performing two distributions at 15-30 days apart [67], distributing baits at den entrances [68]. Immunising fox cubs, which account for two thirds of the fox population in spring, is a key factor for OV success. A study conducted during spring 1997 with SAG2 baits showed that two distributions 2 or 4 weeks apart did not improve either the bait uptake or seroconversion rates in adults and in fox cubs. In contrast, a delayed spring distribution during May or June rather than April increased the seroconversion rate in fox cubs [67]. Another study performed under laboratory conditions confirmed that two successive oral vaccinations performed 35 days apart did not significantly improve the seroconversion rate in foxes, since the humoral response after rabies challenge (performed 6 months after vaccination) were already high after one oral administration [45]. This result confirmed that a single dose of SAG2 vaccine allowed the production of memory B cells and a long-term anamnestic response that protected foxes against rabies. Vaccination of fox cubs by distributing the baits at the entrance of dens allowed increasing significantly the immunization rate of cubs (38% versus 17% for controls without distribution at dens). However, since this type of distribution is costly and time-consuming, it was only recommended for restricted areas, where residual foci exist, in addition to the aerial distribution of baits [68]. In France, where different oral vaccines were used [64], the benefit of oral vaccination was obtained after the fourth year of the programme and the highest costs were for preventive vaccination of pets and prevention in humans [69].

### 5.3 Estonia

The number of rabies cases increased from 74 cases during 1995 to 814 cases during 2003 (Figure 3). The red fox and the raccoon dog were the most frequently infected wildlife [49]. The first OV programme was conducted on Vormsi Island (92 km^2^) during spring and autumn 2004. Manual distribution of baits showed the feasibility of OV in this area [70]. During autumn 2005, the first large-scale OV campaign was implemented in the Northern part of Estonia from the Western to the Eastern border, including national islands. From 2006 to 2010, OV campaigns, co-financed by the EU and the Estonian State Budget, were conducted twice a year throughout the Estonian territory. The organization and implementation of these OV campaigns complied with the recommendations of the EU [29]. Baits were dropped at a density of 20 baits/km^2^ using fixed-wing aircraft along parallel flight paths 600 m apart. Flights took place at an altitude of 100-150 m at an average speed of 160-180 km/h. As a consequence of OV, the number of rabies cases dropped dramatically (Figure 3). The last four rabies cases were detected within five kilometres of the Estonian–Russian Federation border in the South-East. No rabies cases were detected since January 2011. The country was recognized as rabies-free during April 2013. According to recommendations of international organisations, all rabies viruses isolated in areas where SAG2 was used were characterized in the EU Rabies Reference Laboratory to differentiate vaccine strains from viruses maintained by wildlife. All field isolates from Estonia belonged to the classical rabies virus (genotype 1) and were closely related [71,72]. Yearly positivity rates for tetracycline ranged from 85% to 93% in foxes and from 82% to 88% in raccoon dogs. Annual immunisation rates assessed through an ELISA test ranged from 34% to 55% in foxes and from 38% to 55% in raccoon dogs [72]. Since 2011, to prevent re-infections from infected neighbouring countries, OV was restricted to buffer zones (total vaccinated area: 9325 km^2^) at the Russian and Latvian borders using a bait density of 20 baits/km^2^. The 2005-2010 total budget dedicated to rabies control (expenses for parenteral and oral vaccines, aerial distribution, surveillance testing, sample collection and awareness campaigns), and prevention (post-exposure prophylaxis costs) amounted to 12 Million Euro [72]. The cost/effectiveness of the strategy using SAG2 vaccine baits has been analysed on the basis of the funding allocated by the European Commission and was demonstrated as more advantageous than those of neighbouring countries using different vaccines [72].

### 5.4 Italy

Italy has been declared rabies-free since 1997. Rabies re-emerged in Italy in October 2008 in the Friuli-Venezia Giulia region in north-eastern Italy, near the Slovenian border [73]. The rabies virus strain isolated was related to the strains detected in Slovenia, Croatia and other West Balkan countries [73]. Slovenia had previously successfully reduced rabies to 2-3 rabies cases/year from 2004 to 2007 after several OV campaigns, but the prevalence of rabies increased in Slovenia during 2008 due to increased infection pressure from Croatia, where no OV of foxes was organized [73]. Nine rabies cases were reported in Italy during 2008 (Figure 3). Three OV campaigns were implemented between January and September 2009 in limited areas in the Friuli Venezia Giulia affected region using SAG2 baits manually distributed at a density of 20 baits/km^2^ [74]. Although these manual bait distribution OV campaigns reduced rabies in the area close to the Slovenian border, rabies infection spread and reached the province of Belluno (Veneto Region) during 2009 and the Autonomous Provinces of Trento and Bolzano between March and August 2010 after the aerial distribution strategy has been established. Therefore, four emergency vaccination campaigns were conducted according to the EU recommendations from December 2009 to December 2010 using aerial distribution by helicopter in a larger area, including recently affected regions [75–77]. During December 2009-January 2010, the vaccinated area consisted of a 50 km buffer around the location of the most southerly and most westerly rabid foxes, and extended to natural or artificial barriers. This campaign covered 8150 km^2^ (altitude < 1000 m) and SAD B19 vaccine baits were distributed (only SAD B19 vaccine was available at that time) (Figure 3). Then SAG2 vaccine baits were used from April 2010 to November 2012, according to their EU authorization and planned availability. During April-June 2010, the vaccinated area was extended westward and covered 27 950 km^2^ (altitudes < 1500 m, then < 2300 m due to the perpetuation of rabies foci at altitudes > 1500 m). Baits were distributed by helicopter at a density of 20-30 baits/km^2^, with a complementary hand distribution in urban areas and small mountain valleys [75–77]. Four ordinary OV campaigns were implemented during spring (May-June) and autumn (November-December), 2011 and 2012. As a consequence of vaccination, rabies cases dropped from 207 terrestrial animal rabies cases during 2010 to one fox case during 2011 (Figure 3), which was detected during February 2011 in Belluno province. No rabies cases were reported during 2012. After the detection of the last rabies case during February 2011, an emergency vaccination near fox dens was implemented in the areas at higher risk during April 2011 in the Belluno province (5408 baits were distributed). The country was declared officially as rabies-free during March 2013. The proportion of foxes with an anti-rabies virus antibody titer > 0.5 IU/mL ranged according to the vaccination campaign from 46% to 78% [78,79]. The vaccination campaigns planned in 2013 and 2014 were limited to 2385 km^2^ in the Friuli Venezia Giulia region at the border from Slovenia. Importantly, all rabies viruses detected in Italy from 2008 to 2011 were analysed and typed as wild-type strains [80].

### 5.5 Finland

Foxes and raccoon dogs are the main reservoirs of rabies in Finland. Oral vaccination campaigns began in 1988 [81]. Finland has been declared rabies free since 1991. To prevent incursions of rabies from Russia, a border vaccination has been implemented since 1991. Until April-May 2011, the vaccinated area was limited to 5000 km^2^ on the south eastern Finish territory along the Russian border. Since September-October 2011, due to an expansion of rabies in Russian Karelia and the occurrence of rabies cases located about 150 km from Finnish border, the vaccinated area was extended up to 10 000 km^2^. SAG2 baits were used from 2010 at a density of 20 baits/km^2^. In addition, a cooperation was established with Russia for vaccination at its borders with Finland (using a different oral vaccine), with a financial support from the EU. In the vaccinated area, no rabies cases were detected in 2011 and 2012. In the vaccinated area, 53% of wild animals were tetracycline-positive in 2011 and 66% in 2012 (until October). Neutralizing antibody titers ≥ 0.5 IU/mL were detected in 39% and 55% of wild animals in 2011 and 2012 (six month follow-up), respectively [81].

## 6. Conclusions

The success of rabies management through OV of wildlife populations has been demonstrated in several European countries. The SAG2 strain provided a safe and potent alternative to other attenuated strains of rabies virus which retain residual pathogenicity. Use of the SAG2 vaccine contributed to the elimination of rabies in Estonia, France, Italy and Switzerland. Importantly, these countries were declared free of rabies after only few years of OV campaigns with SAG2 baits (France also used the recombinant V-RG vaccine) distributed with an appropriate strategy [29]. The excellent tolerance of the SAG2 strain has been confirmed in the field since its first use in 1993. In contrast to other SAD-derived vaccines [8,16,82–84], no safety issue have been reported with SAG2. In particular, no vaccine-induced rabies cases were documented after the distribution of more than 20 millions SAG2 baits throughout Europe.
